# A motivational training program for secondary physical education teachers based on the circumplex model: a study protocol of a randomised controlled trial

**DOI:** 10.3389/fpubh.2024.1461630

**Published:** 2024-08-21

**Authors:** Javier García-Cazorla, Javier Sevil-Serrano, Luis García-González, Ángel Abós

**Affiliations:** ^1^Department of Didactics of Musical, Plastic, and Body Expression, Faculty of Health and Sport Sciences, EFYPAF “Physical Education and Physical Activity Promotion” Research Group, University of Zaragoza, Huesca, Spain; ^2^Department of Didactics of Musical, Plastic, and Body Expression, Faculty of Teaching Training, University of Extremadura, Cáceres, Spain

**Keywords:** circumplex approach, motivating styles, professional development, self-determination theory, psychological needs, motivation

## Abstract

In most self-determination theory (SDT) research, improving (de)motivating teaching styles provides numerous benefits for students and teachers, although there is less evidence of the latter. Although the recent circumplex model provides a fine-grained picture of the different (de)motivating teaching styles (i.e., autonomy support, structure, control, and chaos) that physical education (PE) teachers can use in their lessons, no previous motivational training programs have been based on this model. Moreover, all SDT-training programs have been implemented through different group sessions, but individual sessions have not been delivered. This study outlines the protocol of a motivational training program, derived from the circumplex model, designed to enhance motivating teaching styles (and prevent or decrease demotivating teaching styles) among PE teachers. Consequently, this program seeks to improve motivational variables and influence (mal)adaptive outcomes in both teachers and students. A randomised controlled trial design with a mixed-method approach. At least 16 secondary PE teachers will be assigned to either an experimental group or a control group, together with some of their students. The training program comprises four face-to-face group sessions and two follow-up sessions (one individual and one group session). PE teachers will learn how to support autonomy and provide structure, as well as to be less controlling and chaotic towards students. Over approximately five months, teachers will implement these motivational strategies during their PE classes. Different (de)motivating teaching styles, motivational variables, and (mal)adaptive outcomes will be assessed in both PE teachers and their students at three distinct points: before the training program (T1), during the intervention (T2), and at the end of the intervention (T3). Additionally, two discussion groups involving all experimental PE teachers will be held (one following the training program and another at the end of the intervention). The results from this study could be useful for developing motivational training programs for in-service PE teachers.

**Clinical trial registration**: ClinicalTrials.gov, identifier [NTC06479369].

## Introduction

Physical Education (PE) teachers play a pivotal role in guiding students through their learning process. Drawing on the Self-Determination Theory [SDT; (1)], teachers’ (de)motivating style, referred to as “the interpersonal sentiment and behaviour that teachers rely on during instruction to motivate students to engage in and benefit from learning activities” [(2), p. 94], is a crucial element in the teaching process. Recent research suggests that PE teachers employ a diverse array of teaching behaviours in their educational practise (3). Autonomy, competence, and relatedness-supportive teaching behaviours (i.e., need-supportive teaching behaviours) have been positively related to students’ autonomous motivation and adaptive outcomes in PE, while the opposite is true for autonomy, competence, and relatedness-thwarting behaviours (i.e., need-thwarting behaviours) (4, 5). Consequently, continuous development teaching (CDT) programs based on SDT (1), have increased in the last years. These SDT-training programs, mainly focused on autonomy-supportive strategies, revealed positive effects on students’ perceptions of (de)motivating teaching behaviours and motivational outcomes (4). Over the past decade, SDT-training programs have also positively affected teachers’ self-perceptions of certain antecedents, support for autonomy and structure, and various motivational and (mal)adaptive outcomes (6). However, additional research is required, as most studies have not focused on reducing need-thwarting behaviours.

Recently, grounded in SDT (1), the circumplex model (7) offers a detailed view of the different (de)motivating teaching styles (i.e., autonomy support, structure, control, and chaos) that teachers can adopt in their classes. This circumplex model delineates eight teaching approaches across these four teaching styles (7, 8). To develop the most effective interventions, it is crucial for researchers to understand the effectiveness of motivational training programs, not only in terms of the four (de)motivating teaching styles but also across the eight specific teaching approaches. Yet, no existing motivational training programs have incorporated this new circumplex approach. This mixed-method study sets out to expand existing knowledge by describing a protocol for a motivational training program based on the circumplex model, aimed at enhancing motivating teaching styles, as well as adaptive outcomes among PE teachers and their students.

According to SDT (1), PE teachers should satisfy the three basic psychological needs (i.e., autonomy, competence, and relatedness) in teaching to experience well-being and feel fulfilled in their jobs. However, it has been established that these needs can also be frustrated (9). Autonomy satisfaction is linked with PE teachers’ sense of making their own decisions and implementing their ideas in lessons, whereas autonomy frustration arises from feelings of compulsion to teach in prescribed ways and experiencing pressure in work-related tasks. Competence satisfaction involves PE teachers’ perception of success and effectiveness in their PE lessons, whereas competence frustration refers to experiencing feelings of ineffectiveness and failure in their teaching-related tasks. Lastly, relatedness satisfaction is experienced by PE teachers when they feel connected and integrated with their colleagues and students, while relatedness frustration occurs when they feel isolated and excluded in their work environment (1, 9). According to SDT, teachers’ need satisfaction and frustration can be influenced by several antecedents, including contextual factors, personal factors, and perceptions of others’ behaviours and motivation (10, 11). Moreover, in alignment with SDT, these PE teachers’ need-based experiences significantly influence their well-being and play a crucial role in the (de)motivating teaching styles they adopt during PE lessons. Previous SDT-based research (12) indicates that teachers’ need satisfaction is positively associated with different adaptive outcomes (e.g., well-being, job satisfaction, engagement, etc.) and need-supportive teaching behaviours towards students. However, teachers’ need frustration has been positively related to maladaptive outcomes (e.g., distress, burnout, etc.) and need-thwarting teaching behaviours towards students. Therefore, addressing certain antecedents of (de)motivating teaching styles could enhance teachers’ need satisfaction and reduce their frustration, consequently facilitating the adoption of motivating teaching behaviours.

The circumplex model offers a deeper and more detailed perspective of the four teaching styles a PE teacher can employ in their lessons (7). These styles are categorised along two axes: one horizontal, indicating whether the style supports or thwarts students’ needs, and one vertical, reflecting the degree of the directiveness exhibited by the PE teachers. Each teaching style is further divided into two distinct approaches, culminating in a total of eight specific teaching approaches (7).

On the one hand, the first motivating teaching style, characterised by low directiveness and high need support, is termed autonomy support. PE teachers who demonstrate a tone of receptivity and flexibility to accommodate the preferences and interests of their students provide an autonomy-supportive environment (13). Autonomy support can emerge through a participative (i.e., PE teacher provides students with choices and decision-making power) and attuning teaching approach (i.e., PE teacher fosters students’ interests, accepts expressions of negative affect, and explains the relevance of each activity performed). The second motivating teaching style, characterised by high directiveness and high need support, is termed structure (7). Structure involves PE teachers adopting attitudes oriented towards progress and process, always considering each student’s ability levels and the needs (13). The structuring style is displayed by a guiding (i.e., PE teacher provides students with helpful guidelines and encouragement for successful task completion) and a clarifying teaching approach (i.e., PE teacher communicates the goals and expectations of the lessons to the students) (8).

On the other hand, the first demotivating teaching style, characterised by high directiveness and high levels of need-thwarting, is termed control. It refers to those PE teachers who exert pressure on students to think, feel, and behave in specific ways (13). This controlling style can be expressed by a demanding (i.e., PE teacher imposes mandatory actions on their students and administers punishment or threats if they fail to comply) and a domineering teaching approach (i.e., PE teacher uses manipulative strategies such as inducing shame, disapproval, or even humiliation to comply with their requests) (14). The second demotivating teaching style, characterised by low directiveness and high levels of need-thwarting, is termed chaos. It refers to those PE teachers who adopt a *laissez-faire* approach, characterised by their unpredictable and inconsistent behaviour (13). The chaotic style is expressed by an abandoning (i.e., after multiple failed attempts, the PE teacher resigns and leaves the students to fend for themselves) and an awaiting teaching approach (i.e., PE teachers do not plan lessons extensively as they prefer to wait and see how things unfold) (7).

Previous studies based on SDT and the circumplex model (14, 15) have shown that teachers whose needs are satisfied implement autonomy-supportive (i.e., participative and attuning approaches) and structuring styles (i.e., guiding and clarifying approaches) in their PE classes, while those teachers whose needs are frustrated use controlling (i.e., demanding and domineering approaches) and chaotic styles (i.e., abandoning and awaiting approaches). According to SDT, adopting these (de)motivating teaching styles/approaches by PE teachers may lead to various motivational consequences for students.

Grounded in the circumplex model, a growing body of research examines the relationship between (de)motivating teaching styles/approaches and students’ motivational outcomes. For example, Burgueño et al. (14) demonstrated that students’ perceptions of autonomy-supportive (i.e., participative and attuning) and structuring (i.e., guiding and clarifying) styles by PE teachers are positively related to students’ need satisfaction, but the clarifying approach is also negatively associated with students’ need frustration. Diloy-Peña et al. (16) also showed that those students who perceived autonomy-supportive and structuring styles and approaches reported higher values in positive PE experiences, learning in PE, and intention to participate in physical activity (PA). Conversely, Abós et al. (14) showed that controlling (i.e., demanding and domineering approaches) and chaotic (i.e., abandoning and awaiting approaches) styles are positively associated with students’ need frustration but that the domineering and abandoning approaches are also negatively associated with students’ need satisfaction. Additionally, comprehensive SDT-based research in PE indicates that students’ need satisfaction is positively related to autonomous motivation and positive behavioural, affective, and cognitive outcomes, whereas students’ need frustration is positively related to controlled motivation, amotivation, and various maladaptive outcomes (4). Consequently, given the associated benefits for students, SDT-training programs aimed at improving PE teachers’ motivating teaching style have increased in recent years.

SDT-training programs have predominantly concentrated on autonomy-supportive strategies, revealing positive effects on students’ perceptions of their PE teachers’ (de)motivating teaching styles/approaches, as well as on motivational outcomes in PE lessons (4). A previous review of SDT-training programs also suggested that PE teachers can benefit from participating in these programs, although more research is needed to examine their effects on a wide range of outcomes (6). However, there are very few SDT-training programs that examine their effects on both students and teachers. For example, Cheon et al. (17) showed that PE teachers who participated in an eight-hour, three-session face-to-face SDT-training program, focused on providing structure in an autonomy-supportive way, showed improvements across all assessed variables (i.e., teacher-reported autonomy support and structure, teaching efficacy, intrinsic instructional goals, harmonious passion, job satisfaction, and relationship satisfaction with students). Moreover, students also perceived improvements in autonomy and structure support, autonomy and competence satisfaction, and outcomes such as classroom engagement, skill development, anticipated PE performance, and future intention to do PA. Notably, in most of these SDT training programs, the effects on teachers have only been assessed after implementing the strategies with the students (18). Gaining insight into teachers’ perspectives both before the training, immediately after the SDT-training program, and upon completion of the entire intervention could enhance the program’s acceptance, sustainability, and scalability. To achieve this, employing a qualitative methodology (in addition to quantitative) could build on existing findings, providing greater justification for the results obtained.

To our knowledge, no previous motivational training programs have been designed based on the circumplex model. This model can guide the teaching approaches associated with each of the (de)motivating styles and provide a better understanding of the potential effects of the program on each of the eight teaching approaches. In the educational domain, only three programs to date have examined the effects of SDT-training programs on (de)motivating teaching styles, using the Situations-in-School Questionnaire (SIS; an instrument to assess the eight teaching approaches proposed by the circumplex model) (17, 19, 20). However, none of these programs have specifically examined the effects of all eight teaching approaches proposed by the circumplex model. In addition, few SDT-training programs have focused on reducing controlling and chaotic teaching styles. As teachers may combine need-supportive and need-thwarting approaches (3), it seems necessary for these programs to also focus on reducing these behaviours.

The characteristics, content, and implementation mode of the SDT-training programs also appear to be crucial factors to consider. To date, all SDT-training programs have been implemented through different group sessions with PE teachers. However, different person-centred studies have indicated that each PE teacher may exhibit a very different (de)motivating teaching style profile (3). For instance, one teacher might employ both autonomy-supportive and structuring styles alongside a controlling style. Conversely, another teacher might use autonomy-supportive behaviours while lacking structure, resulting in a chaotic classroom environment. Therefore, it seems necessary for at least part of the motivational training program to be tailored to each teacher’s (de)motivating teaching profile and personal characteristics. The use of observational methodology in teacher’s classes has emerged in recent years as a solution to provide constructive and individual feedback through videos (21). However, to date, it has only been used in SDT-training programs to examine intervention fidelity and/or to assess possible changes in the (de)motivating teaching styles (6). Combining a brief initial theoretical component with a more extensive practical part (i.e., microteaching) has been previously identified as essential for applying what has been learned in contexts as close to reality as possible (18). Ultimately, the adoption of a congruent style, where trainers implement the program using (de)motivating teaching styles, has also been positively perceived by PE teachers. This approach allows them to observe real-life examples of how to implement these strategies effectively (18). Moreover, in line with SDT, it could lead to an immediate effect on the teachers’ need satisfaction during the training, which has been positively associated with effectiveness and feasibility beliefs in terms of autonomy support and structure, as well as teachers’ intentions to apply the proposed strategies (22).

Finally, while previous studies have considered gender as a covariate to analyse the effects of SDT-based interventions, gender differences in study variables are seldom reported for either teachers or students. Given prior SDT-related research indicating that (de)motivating teaching behaviours may be perceived differently by male and female students (5) and male and female teachers (15), it is crucial to determine whether the intervention is equally effective for both genders to mitigate any potential gender-related inequities.

To extend previous knowledge, this mixed-method study describes the protocol of a motivational training program, based on the circumplex model, aimed at improving (de)motivating teaching styles/approaches among PE teachers. The first hypothesis suggests that the features of the motivational training program will be positively perceived by PE teachers (H1). We also hypothesise that experimental female and male school teachers will perceive improvements in their students in several antecedents, autonomy and competence satisfaction/frustration at work, (de)motivating teaching styles/approaches, and (mal)adaptive outcomes at least at the end of the intervention implementation (H2). Finally, both boys and girls from the experimental groups will perceive improvements in their PE teachers’ (de)motivating teaching styles/approaches, autonomy and competence satisfaction/frustration in PE, and adaptive outcomes in PE lessons (H3).

## Materials and methods

### Context, design, and randomisation

This study will be carried out in a northeast region of Spain. PE is mandatory for every secondary school student in Spain. Each student receives two 50-min coeducational PE lessons per week. Spanish Secondary PE teachers are expected to teach between 18 and 21 h per week. Typically, the annual teaching plan of PE teachers includes approximately six to eight distinct teaching units per year. These units encompass various content types such as individual, cooperative, and interactive sports and games, as well as body expression, health-related fitness, and outdoor activities, all outlined in the PE curriculum. The academic year lasts from September to June, divided into three terms, each separated by a holiday period (i.e., Christmas and Easter).

A randomised controlled trial design with a mixed-method approach will be carried out. PE teachers agreeing to participate in the trial will be randomly assigned to either the experimental or the control group. Randomisation will be conducted using the digital tool available at https://echaloasuerte.com/. To prevent contamination of the experimental condition, schools will only participate in one group (i.e., experimental or control). Subsequently, at least two groups of students from each PE teacher will be randomly selected.

**Figure 1:**
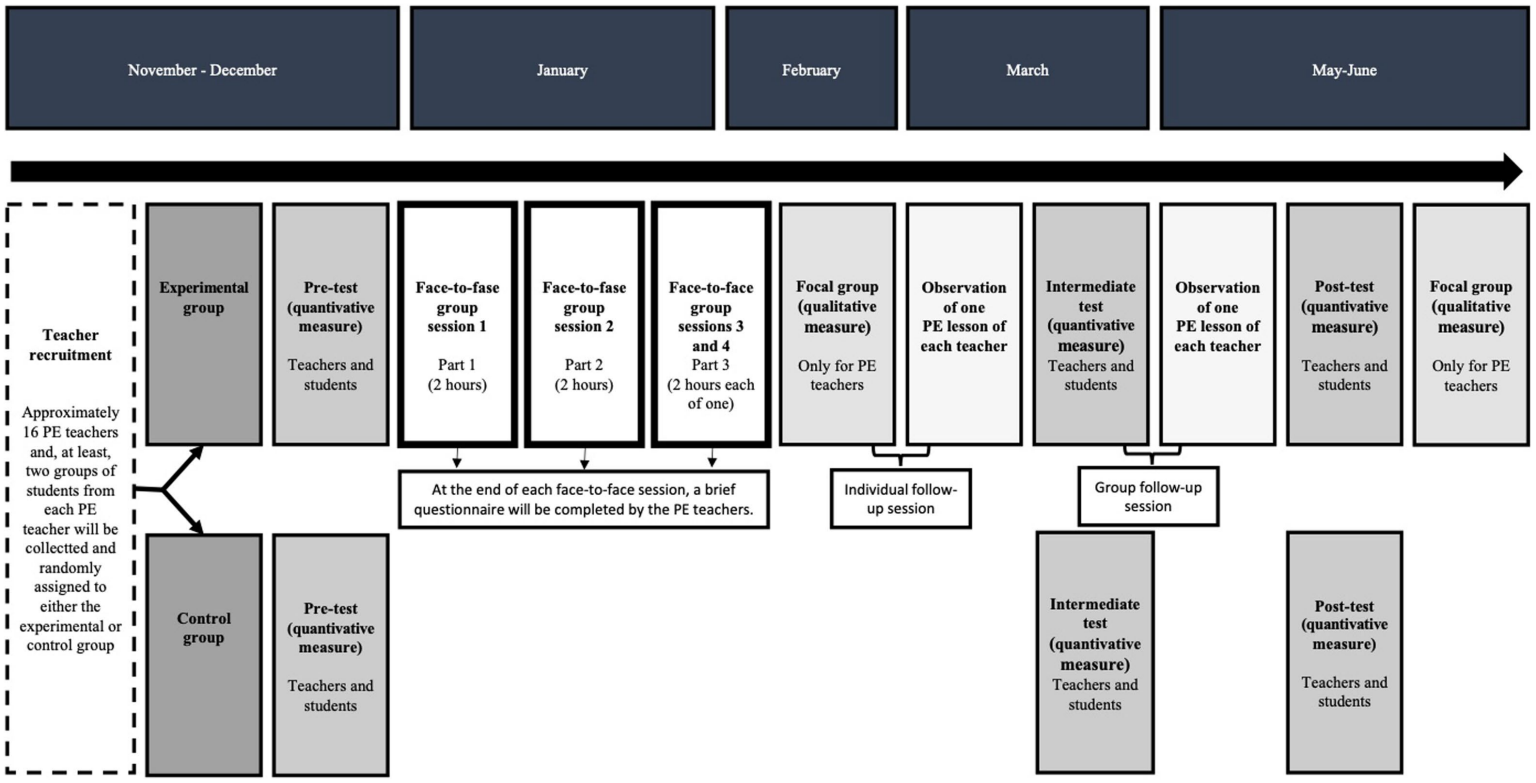
Characteristics of the training program and timeline for data collection. * The variables assessed in PE teachers and students are detailed in the corresponding section; ** the training program has two follow-up sessions (individual and group).

This study will comprise two phases: 1) a teacher-training phase and 2) an implementation phase with students. It should be noted that the training program will continue with some individual and group sessions during the implementation phase. As teachers’ and students’ perceptions of (de)motivating teaching styles and approaches and other study variables require several months for greater accuracy of their perceptions, the training program will not start until the second term of the academic year (for more details, see Figure 1).

The study was approved by the Ethics Committee of the University of Extremadura (code: 153/2022) and follows all ethical procedures established in the Declaration of Helsinki.

### Sample size calculation

The sample size for this intervention-based study was calculated using R Studio to ensure adequate power in the detection of potential statistically significant effects. Considering a multi-level design nesting centre, PE teacher and group, the sample size calculation was grounded on an anticipated effect size of 0.5, reflecting a moderate impact of the intervention. The power of the study was set at 80% with a significance level (alpha) of 0.05, aligning with common practises in educational research (23). Acknowledging the inherent structure of this study, an Intra-Class Correlation (ICC) of 0.10 was assumed based on similar educational settings (24). This ICC estimate accounted for the expected homogeneity within the three levels (class group within the PE teacher, PE teacher within the centre). To accommodate potential participant dropout, particularly among students, we incorporated a 40% anticipated dropout rate into our calculations [e.g., (25)]. The minimum sample size was adjusted to 210 students across 15 PE teachers, averaging 14 students per teacher. This adjustment ensures that our study maintains sufficient statistical power even in the face of anticipated losses, thereby safeguarding the integrity and validity of our findings.

### Participants and recruitment

At least 16 secondary PE teachers, eight in the experimental group and eight in the control group, along with their respective students, will be expected to participate in this study. The maximum number of participating teachers will be capped at 20, due to the limited human resources of the research team. PE teachers will select at least two classroom groups comprising at least 14 students each to invite to participate in this study. Eligible students will be those aged 12 to 17 years in secondary schools. Participation will be voluntary and anonymous.

Various social media platforms (i.e., Instagram, Twitter, and WhatsApp) and other communication methods (i.e., email) will be used for teachers’ recruitment. An informative poster will be launched, detailing the target sample, content, aims, and training program dates. This poster will also include two links (QR code): one with a document providing further information and another to registration through a brief Google Forms questionnaire. Regarding the additional information document, it is important to note that it will detail a more comprehensive overview of the objectives, the different phases of the program, inclusion criteria, the requirements that each teacher must accept to participate, and the teaching skills that will be developed during the training program. Teachers with further inquiries can contact the research team via email or phone. The registration period will last approximately 21 days.

Once the interested PE teachers have registered, various inclusion criteria will be considered for the final selection: 1) Being an in-service PE secondary school teacher for the entire academic year; 2) Attending 100% of the training program sessions; 3) Filling in a short questionnaire at the end of each session of the training program, as well as completing questionnaires of the study variables three times; 4) Allowing the recording of two PE lessons; 5) Participating in two focus groups, one at the end of the training program and one at the end of the study, and 6) Not participating in other training sessions related to PE instruction during the program. Moreover, the inclusion criteria for students will be: 1) Authorisation from parents or legal guardians; 2) Completion of questionnaires of the study variables three times; 3) Regular participation in PE lessons.

### Measures

#### Questionnaires

The following PE teachers’ variables will be measured using Google Forms before the training program (T1), as well as during (T2) and at the end of the implementation of the intervention with their students (T3) (see Figure 1):

##### Socio-demographic variables

Age, gender, teaching experience, type of school (public or private), and school location (rural or urban) will be self-reported by teachers.

##### (De)motivating teaching styles and approaches towards students

To assess self-reported (de)motivating teaching styles toward students, the Spanish version of the SIS in Physical Education [SIS-PE; (14)] will be used. The SIS-PE comprises 12 typical teaching situations consisting of four items each (i.e., 48 items). Autonomy-supportive items are categorised into participative (four items) and attuning (eight items) approaches. Structure items are operationalised into guiding (seven items) and clarifying (five items) approaches. Control items are divided into demanding (seven items) and domineering (five items) approaches. Chaos items are operationalised into abandoning (eight items) and awaiting (four items) approaches. For instance, this steem phrase “At the start of class…” is followed by four items: “you can explore students’ prior knowledge of the topic (attuning),” “set up a class clearly and straightforwardly (clarifying),” “demand application of what is taught (demanding),” or “just begin and let the class evolve (awaiting).” It should be noted that, as teachers’ perceptions of their (de)motivating teaching styles could be different according to the classroom group, teachers will have to answer the questionnaire taking into account the groups of students selected for the study. Teachers’ responses will be assessed using a 7-point Likert scale ranging from 1 (*Does not describe me at all*) to 7 (*Describes me perfectly*).

##### Autonomy and competence satisfaction and frustration at work

To assess PE teachers’ perceptions of autonomy and competence satisfaction and frustration at work, the Spanish version of the Basic Psychological Needs at Work Scale for in-service teachers (26) and the Basic Psychological Need Satisfaction and Frustration Scale (27) will be used, respectively. Four of the six factors of these scales will be assessed, except for relatedness satisfaction and frustration. Both scales are preceded by the stem “In my job as a PE teacher…” and assess autonomy satisfaction (Four items; e.g., “My job allows me to make decisions”), autonomy frustration (Four items; e.g., “I feel that most of the things I do in my job, I do them because I have to do them”), competence satisfaction (Four items; e.g., “I have the ability to do my job well”), and competence frustration (Four items; e.g., “I have serious doubts that I can do in my job well”). Teachers’ responses will be assessed using a 5-point Likert scale ranging from 1 (*strongly disagree*) to 5 (*strongly agree*).

##### Job satisfaction at work

Teachers’ perceptions of job satisfaction at work will be assessed using a Spanish translation (28) of the Teacher Job Satisfaction Scale [TJSS; (29)]. This four-item scale includes a single factor (e.g., “I enjoy working as a teacher”). Teachers’ responses will be registered on a 6-point Likert scale from 1 (*strongly disagree*) to 6 (*strongly agree*).

##### Emotional exhaustion at work

Teachers’ emotional exhaustion will be assessed using the Spanish version of the Maslach Burnout Inventory-General Survey (30). In line with other studies on PE teachers (31), only the five items assessing the exhaustion factor will be used in the present study (e.g., “I feel burned out from my work”). Teachers’ responses will be reported on a 7-point Likert scale from 0 (*never*) to 6 (*every day*).

##### Job performance

Teachers’ perceptions of their professional performance will be assessed using the following sentence: “Rate your satisfaction with your professional performance this academic year,” which has been previously used in other studies (32). Teachers’ responses will be provided on a 9-point Likert scale, ranging from 1 (*non-existent*) to 9 (*excellent*).

##### Quality of the training program

Consistent with previous studies (18, 33, 34), a short paper-and-pencil questionnaire will also be applied immediately after each session of the training program to gain insight into their content. Questions will be related to: (1) interaction, (2) innovation, (3) interest, (4) intelligibility, (5) essentiality, (6) practical usefulness, (7) feasibility of the motivating strategies, (8) intention to implement the motivating strategies, (9) the extent to which one would recommend the training to others, (10) perceived changes in their (de)motivating styles, and (11) overall satisfaction. This questionnaire will be rated on a 5-point Likert scale from 1 (*totally disagree*) to 5 (*totally agree*), except for the last question, in which teachers will rate the overall satisfaction on a scale from 1 to 10. Lastly, in an open-ended question, teachers will be able to detail the strengths and areas for improvement in each session to make slight adjustments to the training program in their future implementation.

Like their teachers, students will fill out the following questionnaires before the training program (T1), as well as during (T2) and at the end of the implementation of the intervention by the PE teachers (T3) (See Figure 1). Depending on the protocol of each school, these questionnaires will be completed in paper-and-pencil format or using Google Forms in a quiet classroom environment. The PE teacher will not be present when their students complete the questionnaires to avoid response bias. In this sense, a member of the research will help the students with any doubts.

##### Socio-demographic variables

Age, gender, and school grade level will be self-reported by students.

##### (De)motivating teaching styles and approaches

To assess students’ perceptions of (de)motivating teaching approaches of their PE teachers, the Spanish version of students of the Situations-in-School Questionnaire in Physical Education [SIS-PE; (14)] will be used. The only change in the instrument compared to the teachers’ instrument is the structure of the sentences, as they are written from the students’ perspective (e.g., “Your teacher invites you to suggest a set of norms or rules”).

##### Autonomy and competence satisfaction and frustration in PE

To assess students’ perceptions of autonomy and competence satisfaction in PE, the Spanish version (35) of the Basic Psychological Need Satisfaction and Frustration Scale (27) will be used. Four of the six factors of these scales will be assessed, except for relatedness satisfaction and frustration in PE. Preceded by the stem “In my PE lessons…,” the 16 items (four items per factor) assessing autonomy satisfaction (e.g., “I feel I have been doing what interests me”), autonomy frustration (e.g., “I feel pressured to do too many tasks”), competence satisfaction (e.g., “I feel I can complete difficult tasks”), and competence frustration (e.g., “I feel like a failure because of the mistakes I make”) are presented. Items will be assessed using a 5-point Likert scale ranging from 1 (strongly disagree) to 5 (strongly agree).

##### PE experiences

In line with previous research (16), students’ perceived experiences in PE classes will be assessed using the question: “What are your experiences in PE lessons like?” The response possibilities were: (1) *very bad*, (2) *bad*, (3) *neutral*, (4) *good*, and (5) *very good*.

##### Perceived learning in PE

In line with previous research (16), students’ perceptions of learning in PE will be assessed using the question: “How much do you learn in PE?” The response possibilities will be on a scale from 1 (*nothing*) to 5 (*a lot*).

##### Intention to be physically active

Students’ perceptions of intention to participate in PA will be assessed using three items (e.g., “I intend to do active sports and/or physical activities during my leisure time in the next 5 weeks…”) of the Spanish version of the Theory of Planned Behaviour Questionnaire (36). This is a 5-point Likert scale ranging from 1 (*strongly agree*) to 7 (*strongly disagree*).

#### Observation

##### Observed (de)motivating teaching styles and approaches

Before the study, two raters with expertise in PE teaching instruction and the circumplex model will be trained in how to code (de)motivating teaching styles and approaches during PE using a Spanish translation of the SIS-PE-Coder, a new observation instrument with good reliability and internal validity (37). Following Van Doren et al. (37) procedure, two randomly selected five-minute videos will be coded to represent the beginning, middle, or end of the lesson during six meetings. Before the final meeting, each expert will independently code an entire lesson. Interobserver reliability will be determined through Cohen’s Kappa, using the following formula: agreements / (agreements + disagreements) × 100.

Consistent with the teachers’ version of the SIS-PE, the SIS-PE-Coder consists of 41 items, of which four items represent the participative approach, five items the attuning approach, six items the guiding approach, five items the clarifying approach, five items the demanding approach, seven items the domineering approach, five items the abandoning approach, and five items the awaiting approach. The coder will be prompted to assess each teaching behaviour from the students’ perspective, as specified by the statement: “If you were a student in this PE class, you would believe that the PE teacher…” Each item will be coded on a 7-point Likert scale, ranging from 0 (*does not display this behaviour*) to 6 (*perfectly displays this behaviour*). These two raters will record two classes per experimental group teacher at two different moments of the implementation phase (before the second and third quantitative measures; see Figure 1). Items will be coded at 5-min intervals (18). For every lesson, interval scores will be added to create a sum score for each teaching behaviour throughout the lesson. This sum will be divided by the number of coded 5-min intervals. Subsequently, scores for (de)motivating teaching styles and the eight approaches will be generated by averaging the scores of the individual items corresponding to each of the four styles and eight teaching approaches. The aim of the recordings will be not only to assess the fidelity of the intervention but also to provide opportunities for teachers’ self-assessment, as well as co-assessment by a member of the study.

For the observational analysis recordings, various professional video cameras will be used, as well as microphones connected to both the camera and the PE teacher who will be conducting the lessons. The iMovie (IOS) program will be used to digitise the video from the start to the end of the PE class.

#### Focus groups

Two discussion groups with all PE teachers will be held throughout the study (See Figure 1). Firstly, one focus group will be held immediately after the end of the fourth session of the training program. The main themes covered in the focus groups will be (1) the content of each session of the program (i.e., theoretical background, design of motivational strategies in different teaching units, and implementation of different PE lessons), (2) the didactical approach (e.g., images, videos, practical examples, formative assessment, and interactive exercises) and their perception of the trainers’ (de)motivating teaching style (i.e., congruent teaching), (3) perceived changes in beliefs about (de)motivating teaching styles, the satisfaction of their basic psychological needs, and (de)motivating teaching styles towards students, and (4) overall assessment of the training (e.g., innovation, practical usefulness, feasibility of the motivating strategies, intention to implement the motivating strategies, satisfaction, etc.). This last question will complement the short questionnaire completed by the PE teachers at the end of each training program session (see above).

The second focus group will take place at the end of the intervention, coinciding with the completion of the teachers’ and students’ questionnaires in the post-test (T3). This will help to determine the teachers’ perception of the implementation phase, as well as of the different study variables. The main themes covered in the focus groups will be (1) the follow-up of the training program (e.g., individual and group sessions), (2) perceived changes in beliefs about (de)motivating teachers’ styles, satisfaction of their basic psychological needs, (de)motivating teaching styles towards students, and job satisfaction, emotional exhaustion, and job performance.

Both focus groups will be facilitated by a female with expertise in PE teaching instruction, the SDT framework, and qualitative methodology. To encourage open communication among PE teachers, the trainers will not be present during the group discussions. Focus group sessions will start with an overview of both the aim and the procedure. The moderator will be supported by a co-moderator, who will manage logistics, record notes, and oversee the recording equipment. Furthermore, to conclude the focus group, the co-moderator will provide a summary of the primary viewpoints and will ask PE teachers whether these perceptions accurately reflect their views or whether they wish to contribute additional insights.

Focus groups will take place in a comfortable, and neutral room, lasting approximately 50 min. All sessions will be videotaped and transcribed to draw conclusions from the discussions (Table 1).

**Table 1:** Summary of the program training sessions.

Face-to-face session 1	Face-to-face session 2	Face-to-face session 3	Face-to-face session 4	Follow-up session 1	Follow-up session 2
The entire group of PE teachers	The entire group of PE teachers	The entire group of PE teachers	The entire group of PE teachers	Individual	The entire group of PE teachers
1.Presentation and getting-to-know-you activity 2.Teaching behaviours of good/bad teachers 3.Explanation of the theoretical backgrounds 4.Design of motivational strategies in PE lessons	1.Summary of the previous lesson 2.Identifying (de)motivating teaching behaviours with real videos 3.Design of specific motivational strategies in different teaching units	1.Application of the motivational strategies in two PE lessons of a simulated real-life situation and subsequent reflection	1.Application of the motivational strategies in one PE lesson of a simulated real-life situation and subsequent reflection 2.Summary of the key points of the four previous sessions	1.Observation of the PE teachers’ real classes and subsequent constructive feedback	1.Observation of the PE teachers’ real classes and subsequent constructive feedback

#### Teachers’ training program and intervention implementation with students

The intervention will comprise two phases in the experimental group: 1) a teacher-training phase (four face-to-face sessions and two follow-up sessions) and 2) an implementation phase where teachers will implement the strategies with the students (see Figure 1).

##### Teachers’ training program in the experimental group

The first part of the training program will last 4 weeks, a total of 8 hours. All PE teachers will participate in four weekly face-to-face group sessions, each lasting 2 hours, scheduled from 17:00 to 19:00. Grounded in the circumplex model, the sessions aim to increase autonomy support and structure, while reducing controlling and chaotic approaches towards students. The training will be delivered by two members of the research team who are experienced in SDT-training programs for PE teachers.

The program incorporates strategies from established SDT-based teacher training programs (6, 17, 18, 38). For example, to lead by example (i.e., congruent teaching), trainers provided autonomy support and structure and avoided control and chaos in all the training program sessions. It is worth noting that the pre-test values of teachers’ and students’ perceptions of teaching approaches will allow the design of the training program to be tailored to the needs of the participants, particularly in the individual follow-up session.

The first two-hour face-to-face training session will unfold in a hybrid theoretical-practical workshop format. It will commence with an introductory presentation by the trainers, followed by a brief review of the objectives and contents of the training program. The session will then transition into an autonomy-supportive exercise, where PE teachers will select a “getting-to-know-you” activity (15 min). Teachers should individually identify on a green sticky note some teaching behaviours of a good PE teacher who taught them (e.g., “they allowed us to choose some tasks”) and on a red note those of a bad PE teacher (e.g., “they constantly punished us”). Then, after sticking these notes on the board, teachers will have the opportunity to read and explain their experiences to the rest of the group (15 min). This activity will be linked to the theoretical background (i.e., SDT and circumplex model) that will be used throughout this training program. Thus, through ongoing collaboration and involvement of PE teachers (e.g., “What do you think the need for competence refers to?”), a concise overview of the SDT framework and circumplex model will be provided. This will be done using real-life examples and personal anecdotes shared by both the trainers and the teachers (30 min). Finally, teachers will be encouraged to individually design a series of generic strategies to support autonomy and provide structure, as well as to be less controlling and chaotic towards students in PE lessons (15 min). They will share these strategies with another teacher (15 min) and, finally, in pairs, with the entire group and trainers. This will lead to a collective reflection on the strategies themselves (e.g., “Why do you think this strategy might satisfy the need for autonomy?”, “Could it satisfy or frustrate some other basic psychological need?”) (25 min). At the end of the session, a brief explanation of the next steps and the objectives for upcoming sessions will be given to foster a positive disposition among the teachers. They will also be asked about the teaching units in their annual teaching plans that they must still teach in each of the classroom groups involved in the study (5 min).

The second two-hour face-to-face training session will involve designing motivational strategies in a practical workshop. In the first part of this session, the previous session will be briefly reviewed, recalling theoretical background, identifying (de)motivating teaching behaviours, and reviewing the implementation in their PE lessons of the strategies proposed by the PE teachers in the last session. For this purpose, teachers will actively explain and assess the acquired learning (15 min). Next, some videos of (de)motivating teaching strategies implemented by other secondary PE teachers will be shown. The videos will be selected based on teaching behaviours that have not been detailed by the PE teachers in the previous training session (e.g., autonomy support; “provide an explanatory rationale”). Teachers should identify (de)motivating teaching behaviours depicted in the videos and consider their potential consequences on students’ basic psychological need satisfaction or frustration (25 min). Subsequently, the teaching units mentioned by the PE teachers in the previous training session will be listed. At least two of these teaching units will be chosen to design motivational strategies for small working groups (30 min). A coordinator of each group will present the different (de)motivating teaching strategies of the teaching unit and trainers, and the rest of the teachers will formatively assess the co-created strategies (30 min). Finally, one teacher from each group will be offered the opportunity to teach one of the lessons of these teaching units to their colleagues, integrating at least two strategies from each (de)motivating teaching behaviour. The other teachers in the group will assist the volunteer teacher in designing the class.

The third two-hour face-to-face training session will involve applying and receiving the strategies learned throughout the training program in a simulated real-life situation (i.e., PE lesson) with the other participating teachers and other volunteers. One teacher from each group will teach one of the lessons of these teaching units using (de)motivating teaching behaviours (45 min per teacher). Afterwards, the trainers and the other PE teachers will provide a formative assessment of positive strengths and areas for improvement (15 min after each class). Finally, another teacher will be encouraged to implement a lesson from the teaching unit of their annual teaching plan, with the help of the research team via Google Meet, in the last session of the training program.

The fourth face-to-face training session will continue the practical application of (de)motivating teaching strategies in a simulated real-life situation. This last session is intended to be an example for the rest of the teachers in which a wide variety of (de)motivating teaching strategies frequently appear. This last session will follow the same procedure as the previous class (45 min of class and 15 min of reflection). In the second part of the class, a final in-depth reflection on the first part of the training program will take place, synthesising all the key concepts covered in the initial phase of the training program across the four sessions. Finally, teachers will receive a dossier of (de)motivating teaching behaviours organised in styles and approaches in line with the motivational behaviour change techniques identified by themselves and the trainers during the training (see the section on intervention implementation). Finally, the possibility of creating a WhatsApp group will be offered to facilitate the follow-up of the training program. Teachers will be able to share their progress, ask questions about implementing strategies, etc.

During the implementation phase, there will be one individual and one group follow-up session of the training program (see Figure 1) to monitor the implementation of strategies, give feedback on positive aspects and areas for improvement, and identify potential barriers or challenges encountered in the implementation of motivational strategies during this period. For the individual follow-up session, the trainers will visit each school to observe one PE class with each teacher. Subsequently, a detailed report of their teaching performance will be provided. The report will include: 1) a series of motivational strategies that the teacher used in their class, 2) a proposal of motivational strategies that the teacher could have used in their class, 3) a report of their teaching profile based on the pre-test questionnaire values, including both the teachers’ self-perception and the students’ perceptions, and 4) advice and motivational strategies to improve their teaching profile in that specific class. For the group follow-up session, the objective will be to analyse each teacher’s videos (taken from the first observational measure). The trainers will thoroughly review each video to extract clips showing each teacher using motivating and/or demotivating strategies. After each strategy is presented, a brief discussion will be held with the other teachers to identify strengths and suggestions for possible improvement of these strategies. These follow-up sessions will be individualised and adapted to the needs of each teacher.

### Intervention implementation with students in the experimental group

It should be noted that, although teachers will begin to apply some of the (de)motivating teaching styles and approaches from the first day they attend the training program, they will be implemented with greater variety, frequency, and intensity after the first part of the training (first four face-to-face sessions). The implementation phase will therefore last approximately 5 months, from January to the end of May. The training program received by PE teachers will focus on the motivational strategies proposed by Ahmadi et al. (39). Teachers will be encouraged to implement as many motivational behaviour change techniques as possible in variety, frequency, and intensity in each PE class.

#### Control group

Control group teachers will not initially receive the training program and, as a result, will not intentionally implement any motivational strategy. They will only complete the questionnaires at the same times as experimental group teachers (See Figure 1). Control group teachers will receive the training program after the last study measurement, as well as an extensive final report on their teaching profile and a series of motivational strategies aimed at enhancing their (de)motivating teaching style.

### Analysis plan

#### Quantitative analyses

Firstly, the effects of the four sessions of the training program on the study variables assessed in teachers will be examined. The overall mean for each of the 11 variables perceived by PE teachers about the quality of the training program (e.g., interaction, innovation, interest, intelligibility, etc.) will also be calculated, representing the mean across the different sessions. Repeated-measures analysis of variance (ANOVA) will be used to assess PE teachers’ global appreciation of the 11 variables related to the quality of the training program (e.g., interaction, innovation, interest, intelligibility, etc.) across the training sessions (i.e., within-subject analyses). Accordingly, each training session will be introduced as an independent variable (i.e., within-subject factor), and repeated measures of the PE teacher-related variables will be entered sequentially as dependent variables.

Secondly, the effects of the intervention implementation in PE lessons on the study variables for both teachers and students will also be examined. Levene and Kolmogorov–Smirnov tests will ensure the equality of variances and normal data distribution, respectively (*p* > 0.05). Cronbach’s coefficient will be calculated for each study variable across the three measurements. To examine the effects of the intervention on the study variables, a 3 × 2 (Time x Condition) repeated-measures multivariate analysis of covariance (MANCOVA) will be performed for both teachers and students. Age, gender, teaching experience, type of school (public or private), and school location (rural or urban) will be introduced as covariates among teachers, whereas age, gender, and school grade will be introduced as covariates among students. Subsequently, to examine intragender differences of the intervention on study variables, a 3 × 2 × 2 (Time x Condition x Gender) repeated-measures MANCOVA will be performed for both teachers and students. The same covariates will be entered as in the previous analysis, excluding gender. Multiple paired t-tests with Bonferroni correction will be calculated to determine between-group (i.e., experimental-control group) and within-group (i.e., pre-post) differences. Cohen’s criteria will be used as indicators of small (0.01), moderate (0.06), and large (0.14) effect sizes (40). All statistical analyses will be conducted using IBM SPSS Statistics v.25.0. Finally, a longitudinal structural equation model will be used to analyse the predictive relationships between the study variables, allowing for the observation of potential differences at the three specific times (i.e., pre-test, intermediate-test, and post-test) when data are collected.

#### Qualitative analyses

Concerning the qualitative data, both focus groups will be transcribed and analysed using NVivo Version 11.0 software to organise and classify data efficiently. The data will be analysed using a thematic analysis following the Braun and Clarke (41) phases. First, three researchers will review all the transcriptions independently to gain familiarity with the data. Second, these researchers will select text fragments related to teachers’ perceptions of the effects of the training program and subsequent implementation with students. Finally, after the code review, the final themes and subthemes containing the relevant meanings extracted from the dataset will be further refined. It is expected that a deductive thematic analysis underpinned by the circumplex approach and SDT will be conducted because most of the questions are related to these frameworks. The other two researchers will supervise and share their viewpoints and interpretations to facilitate agreement during the data analysis.

## Discussion

One of the challenges teachers and researchers face is the difficulty of replicating interventions that have shown promising results. This endeavour is often hampered by inadequate reporting of intervention protocols and content. A detailed description of the training program and the subsequent intervention may facilitate scalability in other areas, countries, and contexts. To fill this gap, the present study aims to comprehensively describe the protocol of a motivational training program, based on the circumplex model, aimed at improving the autonomy-supportive and structuring teaching approaches and minimising controlling and chaotic styles among PE teachers.

This study will provide a unique contribution to knowledge in ten key areas: (1) it will be the first motivational training program based on the recent circumplex model, due to the recent and innovative nature of this approach, and the eight teaching approaches proposed by the circumplex model will be assessed using real-life educational situations through the SIS-PE instrument; (2) the quality of the face-to-face training sessions and follow-up sessions of the training program will be examined through short questionnaires at the end of each class, as well as through a focus group with all the PE teachers; (3) the effects of the intervention on a wide range of study variables will be evaluated using a mixed-method approach (i.e., questionnaires and focus groups) in both teachers and students; (4) the effects of the intervention on male and female students and teachers will be examined; (5) not only a post-test, but also an intermediate measure will be used to examine how the study variables vary throughout the program; (6) the training program will not only take place before the implementation of the intervention, but also during the intervention; (7) the training program will include one individual and one group follow-up session in which constructive feedback will be provided, as well as an individualised report; (8) promising strategies that have been shown to be effective in previous SDT-training programs (e.g., congruent style, brief theoretical part, real videos of PE teachers, microteaching, co-creation of teaching strategies, etc.) will be used; (9) the assessment of intervention fidelity through a new validated observational instrument in line with the circumplex model (i.e., SIS-PE-Coder) will be used; (10) motivational behaviour change techniques provided by Ahmadi et al. (39) will be used during the intervention implementation to determine which behaviour change techniques are attributed to the intervention effects.

Likewise, some of the expected results for both teachers and students about the development of this training program will be presented according to the three hypotheses. Regarding the first hypothesis, as promising strategies used in previous SDT-training programs will be used [e.g., (18)], experimental group teachers will perceive the training program positively (e.g., innovation, practical usefulness, feasibility of the motivating strategies, intention to implement the motivating strategies, satisfaction, etc.). Teachers’ feedback will help to modify the training program before it is disseminated to other areas, countries or contexts. This could enhance the training program’s acceptability, sustainability, and scalability.

Concerning the second hypothesis, experimental female and male school teachers are expected to perceive improvements in several antecedents, autonomy and competence satisfaction/frustration at work, (de)motivating teaching styles/approaches, and (mal)adaptive outcomes at least at the end of the intervention implementation with students. According to SDT, teachers are expected to improve malleable antecedents such as (de)motivating teaching style beliefs due to scientific evidence or viewing videos of real classrooms of PE teachers. According to SDT, improving the different antecedents could, in turn, improve autonomy and competence satisfaction/frustration at work (11, 22). The large repertoire of teaching strategies learned during the training program may also enhance teachers’ autonomy and competence satisfaction at work, as well as reduce their autonomy and competence frustration at work, as they will feel they have more resources to cope with their teaching. Finally, according to previous studies in PE teachers, autonomy and competence satisfaction at work could favour greater job satisfaction and job performance (42), as well as greater use of autonomy-supportive (i.e., participative and attuning) and structuring styles (i.e., guiding and clarifying) (14, 15). Conversely, reduction of autonomy and competence frustration at work could favour lower emotional exhaustion (42), as well as a lower use of controlling (i.e., demanding and domineering approaches) and chaotic styles (i.e., abandoning and awaiting approaches) toward students (14, 15).

Finally, regarding the third hypothesis, it is expected that both boys and girls from the experimental groups will perceive improvements in (de)motivating teaching styles/approaches because of the implementation of strategies by their PE teachers over approximately 5 months (4). According to SDT, when students perceive that their PE teachers use autonomy support and structure, they will likely feel autonomy and competence satisfaction. Conversely, if they perceive controlling and chaotic teaching styles, they will likely feel autonomy and competence frustration (4). Finally, it is expected that through the improvement of need-based experiences, students will achieve improvements in affective (i.e., PE experiences), cognitive (i.e., learning in PE), and behavioural (i.e., intention to be physically active) outcomes (4).

### Limitations

Some of the limitations in the development of the teacher training program are as follows. Firstly, the training duration should be kept brief to ensure that PE teachers grasp and internalise the motivational strategies, enabling them to effectively integrate these techniques into their PE classes. Acquiring new knowledge requires dedicating time to learn and assimilate, as well as opportunities to practise and self-and co-assess. Nevertheless, an excessively lengthy training program might discourage PE teachers’ participation. In the scientific literature, these programs typically range from three to 12 h, but there is no consensus regarding the ideal duration. If all the teachers are willing to continue the training program, slightly increasing the number of hours could be considered. Secondly, observation will be used twice to ensure intervention fidelity and provide constructive feedback to teachers during the intervention but will not be used as a complementary measure of students’ and teachers’ perception of (de)motivating teaching styles in the three measures of the study due to lack of human resources. Thirdly, teachers’ perceptions of their beliefs about (de)motivating teaching styles will not be assessed by means of questionnaires due to their length, as well as the absence of validated instruments of the controlling and chaotic styles. Additionally, relatedness satisfaction and frustration at work will not be assessed because the training program will not target teachers within the same school. Additionally, teachers’ perceptions of depersonalisation and reduced personal accomplishment (i.e., burnout factors) will not be assessed using questionnaires due to their length. However, all these variables will be assessed through the focus groups to obtain more information on the effects of the training program and the subsequent intervention carried out. Finally, students’ perceptions of relatedness satisfaction and frustration in PE will not be assessed employing questionnaires due to their length and because the teacher training program was based on the circumplex model, which does not address the need for relatedness. As a final limitation, it is very likely that a post-intervention follow-up measure cannot be carried out the following academic year because in Spain, it is very common for teachers to change schools every year. Therefore, it will only be possible to assess students’ perception of the study variables if they have the same teacher. Similarly, the perception of (de)motivating teaching style might change with different classroom groups.

## Conclusion

The present study presents a comprehensive overview of the protocol for a training program designed for in-service PE teachers, based on the circumplex model, to maximise transparency and replicability. We hope that the motivational training program will help PE teachers support autonomy and structure while minimising the use of controlling and chaotic teaching styles. This, in turn, may lead to an improvement in motivational-related variables and adaptive outcomes both in students and teachers. If the results are promising, this study can drive the professional development of motivational training programs for in-service PE teachers.

## Ethics statement

The studies involving humans were approved by the study was approved by the Ethics Committee of the University of Extremadura (code: 153/2022) and follows all ethical procedures established in the Declaration of Helsinki. The studies were conducted in accordance with the local legislation and institutional requirements. Written informed consent for participation in this study was provided by the participants’ legal guardians/next of kin. Written informed consent was obtained from the individual(s), and minor(s)’ legal guardian/next of kin, for the publication of any potentially identifiable images or data included in this article.

## Author contributions

JG-C: Conceptualization, Data curation, Formal analysis, Funding acquisition, Investigation, Methodology, Project administration, Resources, Software, Supervision, Validation, Visualization, Writing – original draft, Writing – review & editing. JS-S: Conceptualization, Data curation, Formal analysis, Funding acquisition, Investigation, Methodology, Project administration, Resources, Software, Supervision, Validation, Visualization, Writing – review & editing. LG-G: Conceptualization, Data curation, Formal analysis, Funding acquisition, Investigation, Methodology, Project administration, Resources, Software, Supervision, Validation, Visualization, Writing – review & editing. ÁA: Conceptualization, Data curation, Formal analysis, Funding acquisition, Investigation, Methodology, Project administration, Resources, Software, Supervision, Validation, Visualization, Writing – review & editing.
